# Altered Topology of the Structural Brain Network in Patients With Post-stroke Depression

**DOI:** 10.3389/fnins.2019.00776

**Published:** 2019-07-24

**Authors:** Xiaopei Xu, Rui Tang, Luping Zhang, Zhijian Cao

**Affiliations:** ^1^Department of Radiology, The Second Affiliated Hospital of Zhejiang University School of Medicine, Hangzhou, China; ^2^Department of Radiology, The First Affiliated Hospital of Zhejiang Chinese Medical University, Hangzhou, China

**Keywords:** post-stroke depression, brain network, network analysis, small-world, diffusion tensor imaging

## Abstract

There is a pressing need to further our understanding of the mechanisms underlying the depression symptoms in patients with post-stroke depression (PSD) in order to inform targeted therapeutic approaches. While previous research has demonstrated a reorganization in the functional brain network of PSD, it remains uncertain whether, or not it also occurs in the structural brain network. We therefore aim to investigate the structural brain network of patients with PSD as compared to post-stroke non-depression (PSND) patients. In addition, our research considers the relationship between network metrics and functional measurements. Thirty-one PSD patients and twenty-three PSND patients were recruited. All patients underwent MRI and functional assessments, including the Barthel index, mini-mental state examination (MMSE), and Hamilton depression rating scale (HAMD). Diffusion tensor imaging was used to construct the structural brain network and to conduct the subsequent graph theoretical analysis. Network measures were computed and compared between PSD and PSND patients. Associations between functional assessments and network measures were studied as well. We successfully detected increased global and local efficiency in patients with PSD. Regions with disrupted local connections were located primarily in the cognitive and limbic systems. More importantly, PSD patients’ global and regional network measures were associated with depression severity, as measured by HAMD. These findings suggest that disrupted global and local network topologies might contribute to PSD patients’ depression symptoms. Therefore, connectome-based network measures could be potential bio-markers for evaluating stroke patients’ depression levels.

## Introduction

After the initial insult of stroke, stroke patients not only suffer from hemiparesis and cognitive impairments, but also may experience a broad spectrum of emotional disturbances (Mukherjee and Patil, 2011). One of the most commonly developed emotional disorders after stroke, post-stroke depression (PSD) affects the quality of life of one-third of stroke survivors (Lenzi et al., 2008) while also jeopardizing their motor rehabilitation outcomes. Because PSD is so prevalent, this condition requires more attention, and should be treated with caution. However, PSD historically has been considered a prototypical neuropsychiatric manifestation following a major life-changing event, leading to its neglect (Broomfield et al., 2014) in clinical practice. Also problematic, figures vary greatly regarding its prevalence, from 20 to 60% (Paolucci et al., 2005); this variability results from a lack of consensus in diagnostic criteria and depression rating scales, as well as high inter-rater and intra-rater variability (Paolucci et al., 2005). Improved acute management of the disease could help lower mortality in PSD patients. Accordingly, a better understanding of the underlying mechanisms of the disease might assist in developing more accurate early diagnostic criteria and targeted therapeutic methods.

The pathophysiology of PSD currently remains debatable, but two primary views exist: the biological hypothesis and the psychosocial hypothesis (Whyte and Mulsant, 2002). The psychosocial hypothesis emphasizes the impact of social stressors on post-stroke mind status, attributing the primary cause of PSD to psychology (Gainotti et al., 1999). The biological hypothesis, on the other hand, asserts that mood disorders developed post-stroke mainly take root in the impaired neural circuits due to ischemic insults (Beblo et al., 1999). Many imaging studies lend support to this hypothesis, and this body of literature has contributed significantly to the detection of anatomical abnormalities in PSD. Vataja et al. (2001, 2004) repeatedly showed that patients with lesions located in the prefronto-subcortical circuit were more likely to have PSD symptoms. Disruption in the limbic-cortical-striatal-pallidal-thalamic circuit also has been associated with PSD (Terroni et al., 2011). This research consistently suggests that the impaired microstructural integrity of critical neural pathways might confer biological vulnerability for PSD’s onset. Past studies (Yasuno et al., 2014; Ye C. et al., 2016) conducted in PSD patients using diffusion tensor imaging (DTI) align with this hypothesis, indicating an association between microstructural abnormalities in neuroanatomical pathways, and depression severity after stroke. These studies set the foundation for an in-depth investigation into the disrupted neural pathways in PSD, suggesting that looking at PSD from a network perspective in the neuroanatomical area might facilitate our understanding of this disease’s pathophysiology.

Based on past evidence, we form two hypotheses. First, PSD patients will demonstrate significantly lower global and regional topological efficiencies in the structural brain network when compared to PSND patients. Second, the level of network alteration in PSD will be associated with patients’ depression level. To test our hypotheses, we use DTI-based tractography and graph theoretical approaches to investigate differences in the structural brain network of post-stroke patients with and without depression. We also test the relationships between network configuration and functional assessments.

## Materials and Methods

### Participants

This study was approved by the local Institutional Review Board, and written informed consent was obtained from all participants. From 2012 to 2015, 102 patients with first-time ischemic infarct were referred from clinics, and diagnosed by experienced neurologists. To assess the stroke severity of each patient after acute stroke insult, we used the national institute of health stroke scale (NIHSS) and the modified Rankin scale. The classification of acute ischemic stroke was recorded based on the trial of org 10172 in acute stroke treatment (TOAST). Patients were recruited based on the following inclusion criteria: (1) they were over 18 years old; (2) they had been diagnosed with stroke based on the world health organization (WHO) criteria and confirmed by CT/MRI results; (3) they were right-handed; (4) they were within the first 2 weeks following stroke onset; and (5) they presented with modest ischemic insult (mRs ≤ 4), thus having the ability to complete all assessments. Subjects were excluded if they had (1) severe ischemic insult and significant verbal comprehension deficit; (2) a prior history of depression and antidepressant treatment; and/or (3) other neurological or neuropsychological diseases and disorders. Eventually, 18 patients were excluded, leading to a total of 84 patients meeting the criteria and participating in the current study.

All of the subjects were classified as PSD and PSND based on the previously described criteria (Zhang et al., 2014, 2018). The state of depression was diagnosed based on the diagnostic and statistical manual of mental disorders (DSM-IV, fourth edition). The Hamilton depression rating scale (HAMD) of 17 items was used to assess the severity of depression. Patients were categorized as PSD (*n* = 31) if they were (1) diagnosed as having had an acute ischemic stroke based on the above-mentioned criteria; (2) evaluated by two experienced psychiatrists and diagnosed with depression according to the DSM-IV criteria; (3) had a total score of HAMD ≥ 7; and (4) were medication-free during their imaging exam. The location of each PSD patient’s infarct was evaluated and categorized into seven groups, namely basal ganglia, cerebellum, brain stem, frontal lobe, temporal lobe, parietal lobe, and occipital lobe. Following this categorization, we selected the patients’ age, sex, and stroke diagnosis (based on the location and infarct volume), matched stroke patients with a total score of HAMD <7 from the rest of the patient pool, and categorized them as post-stroke non-depression (PSND) patients (*n* = 23). To assess post-stroke disability level, we used the Barthel index (BI). We also administered the mini-mental state examination (MMSE) to evaluate the general cognitive level.

### Image Acquisition

All scans were performed on a 3T MRI scanner (Siemens Verio 3-tesla system; Erlangen, Germany) with an 8-channel SENSE head coil. For each subject, a non-diffusion-weighted image (b_0_) with two averaging and DWIs were acquired using a single-shot echo-planar-imaging sequence with *b*-values = 1000 s/mm^2^ along 59 gradient directions with the following parameters: TR/TE = 6300/95 ms, field of view = 230 mm^2^ × 230 mm^2^, reconstruction resolution = 1.8 mm^2^ x 1.8 mm^2^, slice thickness = 3 mm (no gap), and SENSE factor = 2. For anatomical reference, T1-weighted (T1w) images were acquired using a 3D-MPRAGE sequence with the following parameters: TR/TE/TI = 1900/2.45/900 ms, nominal/reconstruction resolution = 1 mm^3^ x 1 mm^3^ x 1 mm^3^, 176 slices in the sagittal plane, and field of view = 256 mm^2^ x 256 mm^2^.

### Image Processing and Brain Network Construction

The image processing steps required for brain connectivity analysis are explained in detail below.

#### Brain Parcellation

To parcellate the brain into different anatomical regions, we used the automated anatomical labeling (AAL) atlas with 90 cortical and subcortical regions (cerebellum excluded). The mask for each of the brain regions of interest (ROIs) from the atlas was transformed into the individual subject’s native structural MRI space using the following steps. First, the subject’s T1w images were registered to the corresponding DTI images using affine transform with FLIRT. Then, using non-linear transformation with FNIRT, we registered the native space structural images to the ICBM 152 template in the Montreal Neurological Institute’s space (i.e., the same space as the AAL atlas). The inverse of the resulting transformation matrix subsequently was applied to the atlas, thereby bringing all brain ROIs from the AAL atlas into each subject’s native structural MRI space.

#### Diffusion MRI Tractography

For DTI preprocessing, all DWIs were first registered to b0 images to correct for eddy current distortion, and head motion with FMRIB’s Diffusion Toolbox. The diffusion tensor and its associated eigenvectors and eigenvalues were obtained on a voxel-by-voxel basis with the Diffusion Toolkit. To construct the structural connections between the 90 brain regions, DTI-based tractography was performed to track WM fiber tracts using TrackVis^1^ with a fractional anisotropy threshold of 0.2 and a fiber turning angle threshold of 45°.

#### Brain Network

In order to construct the structural brain network, a connectivity matrix has to be estimated to describe the structural connections (i.e., network edges) amongst all brain regions. The connections amongst the 90 brain regions were computed from the WM tractogram using the UCLA multimodal connectivity package. Briefly, the structural connection was estimated by counting the number of WM fiber tracts originating from one region and terminating in another. The fiber count was considered to be the weight of each edge. After repeating this step for all 90 brain regions, an inter-regional undirected weighted network with weighted connections was constructed. To remove the spurious connections and define the network edges, we selected a threshold value for the fiber bundles (Gong et al., 2009; Shu et al., 2011, 2018; Zalesky et al., 2011) and used a minimum threshold of fiber number (w_ij_ = 10, where w_ij_ is defined as the weight of the edge) between two regions. This threshold selection reduced the risk of false positive connections due to noise or limitations in the deterministic tractography; simultaneously, it ensured that the size of the largest connected component in the networks was observed across all controls. We also tested the effects of different thresholds on the network analysis by setting threshold values of w_ij_ ranging from 5 to 15, and this thresholding procedure did not significantly influence our results.

### Brian Connectivity Analysis

We used graph theory to quantify the topology, efficiency, and nodal characteristics of the structural brain network for all cohorts. The individual’s weighted connectivity matrix first was normalized to its largest entry in order to minimize the overall differences in connectivity strength within each subject. Then, for each normalized connectivity matrix, we used the brain connectivity toolbox (Rubinov and Sporns, 2010) to measure small-world properties (clustering coefficient and characteristic shortest path length) and network efficiency (global efficiency and local efficiency), together with the characteristics of each node, including the degree, clustering coefficient, betweenness centrality, and nodal efficiency.

To determine how a network differs from a small-world network, the clustering coefficient and characteristic shortest path length of the current network are often normalized to those of a random network. For each individual brain network, we generated a set of 100 randomized networks with a preserved edge number and degree distribution before calculating the corresponding clustering coefficient and characteristic path length. We considered a network small-world if the normalized clustering coefficient (*γ* = clustering coefficient of current network/clustering coefficient of random network) was much larger than one, the normalized characteristic shortest path length (*λ* = characteristic path length of current network/characteristic path length of random network) was close to one, and the small-worldness (_*σ* = *γ*/*λ*_ ) was larger than one (Watts and Strogatz, 1998).

### Statistical Analysis

Binary variables included sex, history of diabetes mellitus, hypertension, ischemic heart disease, smoking, and presence of infarct in seven brain areas (basal ganglia, brain stem, cerebellum, frontal lobe, parietal lobe, temporal lobe, and occipital lobe). Age, infarct size, and serum total cholesterol were recorded as continuous variables. Demographics were compared between patients with and without PSD using independent samples *t*-test for continuous variables and Fisher’s exact test for proportions. We performed independent-samples *t*-tests to evaluate the group difference in all global and local network measures between patients with and without PSD. To further ensure the robustness of statistical analysis, permutation tests were conducted to assess group differences in global network, and regional measures. For each permutation, individual participants were randomly assigned to one of the two groups with the same size as the original PSD and PSND groups. We then recomputed the mean differences between the two randomized groups. This randomization procedure was repeated 5,000 times, and the 95th percentile points of each distribution were used as the critical values for a one-tailed test of the null hypothesis with a probability of type I error of 0.05. We employed an R statistical software package (version 3.5, R Core Team^2^) and Exact Rank Tests Package for two sample permutation tests. The association between all network measures and all functional assessments were performed using Spearman rank correlation. All analyses were adjusted for age, sex, infarct size, and vascular risk factors (hypertension, diabetes, and serum total cholesterol). For all the statistical analyses described above, we performed a Bonferroni correction for the problem of multiple comparisons, including group comparisons and correlation analysis. All the *p*-values reported were corrected. SPSS 22.0 (SPSS, Chicago, IL, United States) was used for all statistical analyses, and a significance level of *p* < 0.05 was set for all statistical tests.

## Results

### Demographics

Table 1 summarizes patient demographics and the statistical significance of group comparisons. Based on the infarct location, we also classified the stroke patients into three groups: (1) pure cortical infarct (PCI), (2) pure subcortical infarct (PSI), and (3) both cortical and subcortical (BCS) infarct. For PSD patients, there were 5 PCI, 22 PSI, and 4 BCS patients. For PSND patients, there were 1 PCI, 14 PSI, and 8 BCS patients. Patients with PCI seemed more likely to develop PSD; however, this difference was not statistically significant (*p* = 0.096).

**TABLE 1:** Patient demographics.

	**PSD patients**	**PSND patients**	***P*-value**
Sample size	31	23	
Age	64 ± 10	67 ± 12	0.424
Male gender (%)	55	65	0.443
Hamilton depression rating scale	8.5 ± 1.8	3.7 ± 0.8	< 0.001
**Prevalence (%)**			
Diabetes mellitus	16	17	0.866
Hypertension	52	48	0.643
Ischemic heart disease	6	9	0.597
Hyperlipidemia	32	29	0.801
Stroke classification (%)			0.617
Large artery antherosclerosis	35	35	
Small vessel disease	16	9	
Cardioembolism	16	26	
Undetermined	33	30	
**Infarct location (%)**			
Basal ganglia	54	68	0.375
Brain stem	29	11	0.274
Cerebellum	0	11	0.410
Frontal lobe	10	16	1
Parietal lobe	6	11	1
Temporal lobe	6	5	1
Occipital lobe	6	5	1
Infarct size (cm^3^)	5.6 ± 10.1	4.8 ± 9.8	0.793
Infarct sides (L/R)	17/14	12/11	0.650
Smoking	26	39	0.215
Antiplatelets	19	30	0.186
Lipid-lowering drugs	30	26	0.427
Serum total cholesterol (mmol/L)	4.8 ± 1.3	4.4 ± 1.5	0.376
Hemoglobin (g/dL)	13.9 ± 1.4	13.3 ± 1.6	0.176
Serum glucose level (mmol/L)	6.9 ± 3.2	5.8 ± 1.5	0.153

*PSD, post-stroke depression; PSND, post-stroke non-depression.*

There was no significant difference in age, gender, stroke classification, infarct size and location, or baseline vascular risk factors between PSD and PSND patients. Stroke classification, infarct size, and location were not associated with any of the vascular risks. Patients with PSD showed significantly higher NIHSS (*p* = 0.01) and mRs (*p* = 0.02), HAMD (*p* < 0.001), but lower BI (*p* = 0.02). There was no significant difference in MMSE between PSD and PSND patients.

### Global and Regional Network Changes

**FIGURE 1:**
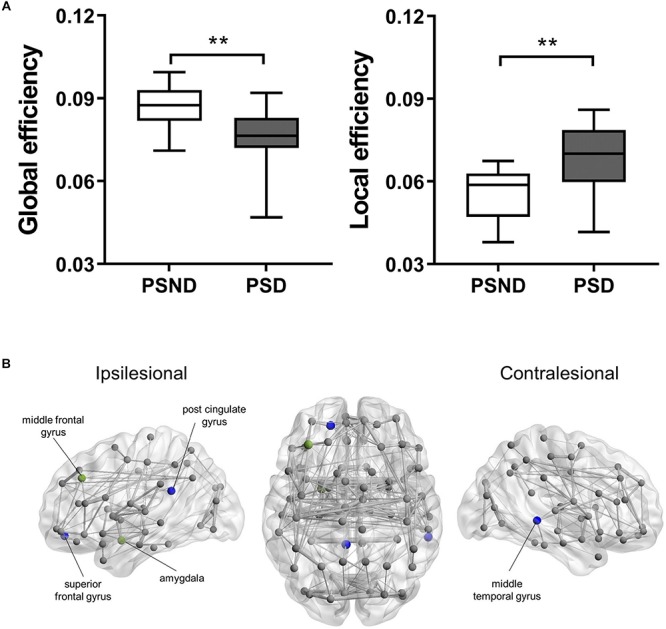
**(A)** The boxplot (mean ± standard deviation) of global and local efficiency of PSD and PSND patients, ^∗∗^*p* < 0.01. **(B)** Illustration of the structural brain network of PSD patients showing brain regions with significant decreases in nodal degree (blue spheres) and nodal efficiency (green spheres) as compared to PSND patients. The width of network edge is weighted by the number of connections. PSD, post-stroke depression; PSND, post-stroke non-depression.

Figure 1 shows the structural brain network of the two patient cohorts. They resemble the properties of the small-world network with a larger clustering coefficient (patients with PSND: 2.81 ± 0.35; with PSD: 2.93 ± 0.46) and the equivalent characteristic shortest path length (with PSND: 1.23 ± 0.03; with PSD: 1.2 ± 0.04) as compared to the random network. Figure 1A shows the global network measures that significantly differ between the two groups. The global efficiency (*p* < 0.001) and local efficiency (*p* = 0.001) of PSD patients’ brain network are lower and higher, respectively, than PSND patients (Figure 1A).

The PSD patients’ nodal degree of ipsilesional superior frontal gyrus (*p* < 0.001), post-cingulate gyrus (*p* < 0.001), and contralesional middle temporal gyrus (*p* < 0.001) were significantly lower than PSND patients (Figure 1B). We also found PSD patients’ nodal efficiency in the ipsilesional middle frontal gyrus (*p* < 0.001), post-cingulate gyrus (*p* < 0.001), and amygdala (*p* = 0.001) to be lower (Figure 1B).

### Relationship Between Network Measures and Functional Assessments

**FIGURE 2:**
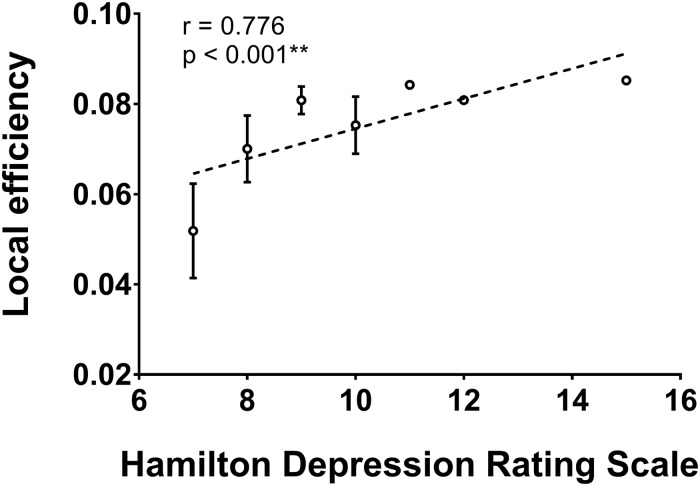
Association between network local efficiency and Hamilton depression rating scale (biomarker of depression severity) for patients with PSD. Error bars represent standard deviations. ^∗∗^*p* < 0.01.

**FIGURE 3:**
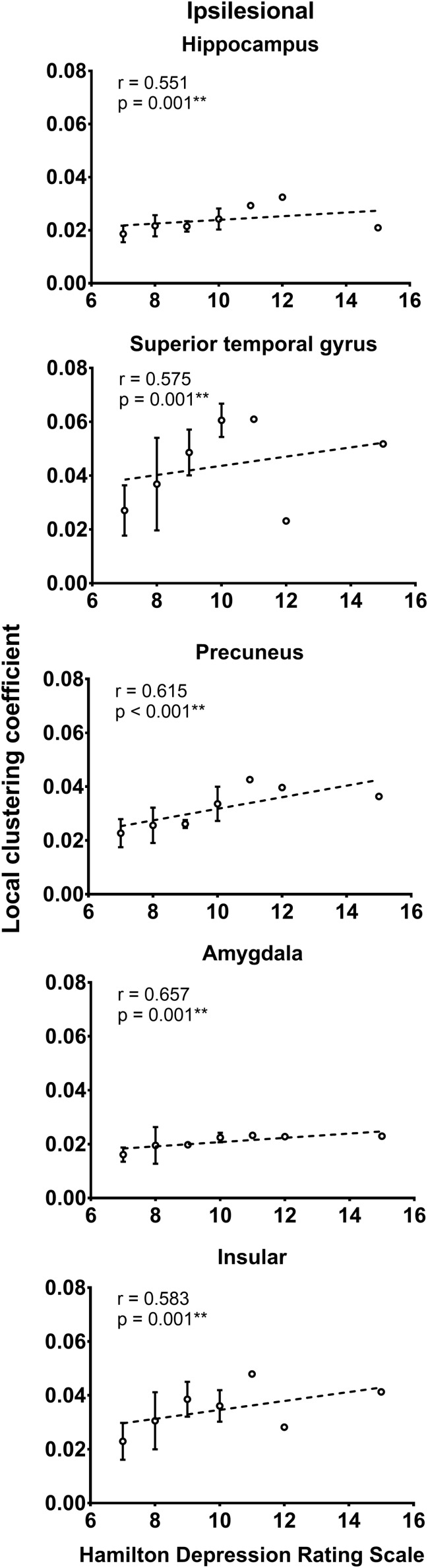
Association between local clustering coefficient and Hamilton depression rating scale (biomarker of depression severity) for patients with PSD. Error bars represent standard deviations. ^∗∗^*p* < 0.01.

**FIGURE 4:**
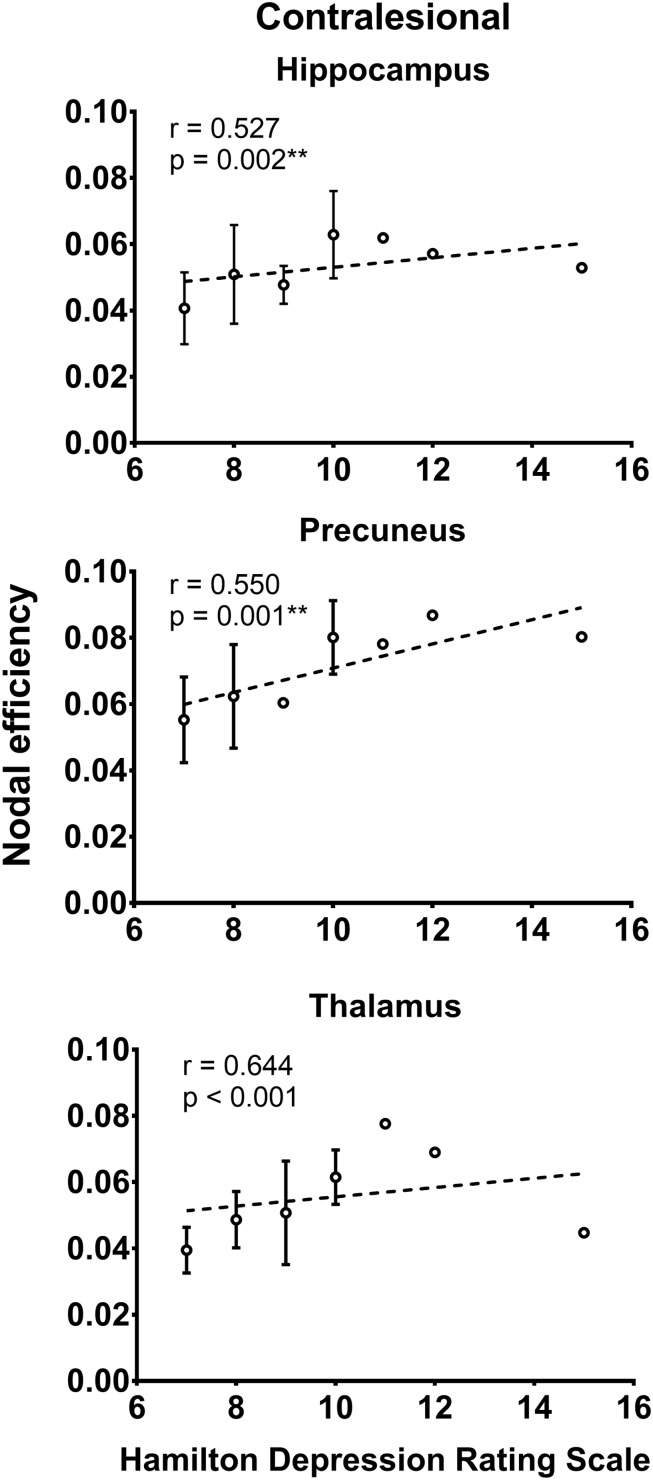
Association between nodal efficiency and Hamilton depression rating scale (biomarker of depression severity) for patients with PSD. Error bars represent standard deviations. ^∗∗^*p* < 0.01.

As shown in Figure 2, the local efficiency (*r* = 0.776, *p* < 0.001) was correlated with HAMD in patients with PSD. In PSD patients, the local clustering coefficient of the ipsilesional superior temporal gyrus (*r* = 0.575, *p* = 0.001), precuneus (*r* = 0.615, *p* < 0.001), hippocampus (*r* = 0.551, *p* = 0.001), amygdala (*r* = 0.657, *p* = 0.001), and insular (*r* = 0.583, *p* = 0.001) was significantly associated with the HAMD (Figure 3). The nodal efficiency of contralesional hippocampus (*r* = 0.527, *p* = 0.002), thalamus (*r* = 0.644, *p* < 0.001), and precuneus (*r* = 0.550, *p* = 0.001) also was related to the HAMD in patients with PSD (Figure 4). There were no significant correlations between other network measures and HAMD, and there were no significant relationships between all the network measures and NIHSS, mRs, and MMSE.

## Discussion

### Shifted Balance Between Functional Integration and Segregation

Brain connectivity analyses have developed rapidly in recent years, repeatedly demonstrating that the human brain is a small-world network. Compared to other forms of networks, including random and lattice networks, small-world networks are superior due to their capacity to support both segregated, and integrated information processing. Functional segregation is responsible for mediating information processes between local communities, while functional integration takes care of global communications. A balance between network segregation and integration is essential to guaranteeing optimal operation for the distributed networks underlying cognitive and behavioral performance. This optimal network configuration can be observed in healthy human brains across different stages of development and across different conditions during task performance (Sporns, 2013). When the balance between network segregation and integration shifts due to disease or disorder, cognitive, and behavioral dysfunction may result.

Compared to PSND patients, patients with PSD showed significantly decreased global efficiency and increased local efficiency in the global structural network organization. While global efficiency quantifies the communication efficiency between long-range connections and promotes functional integration, local efficiency is an indicator of the regional network’s fault tolerance, and reflecting functional segregation (Sporns, 2013). These global network changes in PSD patients suggest that the network configuration became highly clustered (increased local efficiency) and more redundant (decreased global efficiency and increased path length between regions); in other words, it became more like a regular lattice rather than an optimal small-world network (Watts and Strogatz, 1998). Regions that previously were directly connected became indirectly connected, meaning that information being transferred between two regions had to travel through additional intermediaries. In such lattice-like networks, cognitive function becomes compromised because it relies heavily on efficient integrative processing abilities (Bullmore and Sporns, 2012), thus leading to lower performance. This finding aligns with past studies showing that patients with remitted geriatric depression (Bai et al., 2012) have impaired network integration abilities and abnormal regional network properties compared to healthy subjects, primarily in the frontal brain regions. In addition, significantly increased local efficiency was observed in the resting-state functional network of patients with major depressive disorder (Ye M. et al., 2016). Interestingly, while some studies confirm our findings, other structural and/or functional connectivity analyses only partially align with our research. For instance, while treatment-naïve depression patients (Long et al., 2015) had increased local efficiency, they also demonstrated increased global efficiency in the structural network. We thus speculate that the variability in network topological expression found in different studies might result from differing subtypes of depression.

### Impaired Local Network Communication in Multiple Brain Regions

In addition to shedding light on the brain network’s overall architecture, network analysis also could help decipher the interconnected relationships in a specific brain region or between local communities; these clusters formed by regions are either geographically close or serve similar functions (Leergaard et al., 2012). Bearing this in mind, we found that the regions with disrupted regional networks in PSD patients are primarily located in the cognitive and limbic systems, including the superior and middle frontal gyrus, post-cingulate gyrus, middle temporal gyrus, and amygdala.

The superior frontal gyrus and middle frontal gyrus are both involved in emotional disorders and higher cognitive functions (Tucker et al., 1981; Mayberg, 1994). Using modern neuroscience technologies like proton magnetic resonance spectroscopy (Kumar et al., 2002), DTI (Yang et al., 2015b), resting-state electroencephalogram (EEG) (Zhang et al., 2015), and resting-state functional MRI (rs-fMRI) (Vicentini et al., 2017), researchers have found that the frontal lobe of depressive or PSD patients also has white matter biochemical abnormalities, loss of white matter integrity, decreased EEG complexity, and increased functional connectivity. The anatomical basis behind these findings might include the extensive connections between frontal cortex and structures related to emotional behavior, such as the amygdala, basal ganglia, and thalamus (Soares and Mann, 1997). Similarly, the cingulate has been identified as a key area within fronto-limbic networks due to its strong interconnectedness in pathways that are essential for mood and emotional regulation (Drevets et al., 2008). While most mood disorder studies focus on the anterior cingulate cortex, dysfunctions in the posterior cingulate cortex also are related to impaired emotion evaluation, attention, and other cognitive functions (Maddock et al., 2003; Ries et al., 2009). Our findings further support this idea and may encourage the development of effective interventions by identifying potential target regions for deep brain stimulation seeking to normalize network activity (Kringelbach et al., 2011). One of the earliest brain regions discovered to be closely related to emotional disorders, the amygdala has been studied exhaustively; in this regards, both functional and structural connectivity analyses have revealed abnormal regional connections in the fronto-limbic, and cortico-striatal-pallidal-thalamic circuits (Callaghan et al., 2017; Connolly et al., 2017; Delaparte et al., 2017; Jalbrzikowski et al., 2017; Yang et al., 2017). This body of research forms the foundation for novel neuroscience-informed treatment strategies, like amygdala-focused fMRI neurofeedback, while predicting antidepressant treatment outcomes. It thus could contribute to treatment adherence. Meanwhile, our results have demonstrated that network analysis is capable of identifying disconnected regions that might relate to the depression symptoms of PSD patients.

### Associations Between Network Measures and Depression Level

Most importantly, although we were not able to observe any significant relationship between network measures and disability rating scales (e.g., BI, NIHSS, and mRs), we still successfully established the associations between depression level and network metrics using both global and local scales.

The strengths of the current study include the availability of various disability scales and psychological assessments for all subjects. By examining the relationship between global network metrics and depression level measured by HAMD, we found that higher local efficiency was significantly associated with higher HAMD in patients with PSD. As described in the above sections, local efficiency indicates the number of local clusters, and reflects functional segregation. Our results suggest that increased network segregation is associated with more sever depressive symptoms. Although most studies indicate that higher network segregation guarantees a high network fault-tolerance (Sporns, 2013), our findings could indirectly prove that increased network segregation is not always positive. From an economic perspective, brain connections are expensive to build and run, so excessively high network segregation might increase the brain’s overall wiring cost. Other studies also report increased local efficiency in patients with depression symptoms (Long et al., 2015; Ye M. et al., 2016); however, we are the first to demonstrate the significant association between increased local efficiency and HAMD scores.

In patients with PSD, we also observed significant positive associations between depression level and regional network measures in regions like the superior temporal gyrus, precuneus, hippocampus, amygdala, insular, and thalamus. Given that high regional network measures reflect optimal organization in local communication, our findings suggest that PSD induces more efficient regional communications in these regions due to increased regional connections serving as compensatory mechanisms. As shown by previous studies (Ries et al., 2009; Liao et al., 2013; Long et al., 2015; Yang et al., 2017), these regions form the core parts of an effective processing network, which might be impaired in patients with depressive symptoms. Specifically, the amygdala, hippocampus, insular, and thalamus constitute the limbic-cortical-striatal-pallidal-thalamic circuit (Price and Drevets, 2010), and this particular circuit plays an important role in the pathogenesis of depression. Consequently, microstructural changes in the circuit may lead to continually enhanced regional interactions in patients with PSD. As a previous study (Yang et al., 2015a) demonstrated, dysfunctions in the insular, putamen, and superior longitudinal fasciculus are associated with major depression in patients with PSD. Our results align with other network analyses that have demonstrated hyperconnections in certain networks due to a compensatory mechanism in the ventromedial prefrontal network and the salience network of depression (Guo et al., 2013; Wei et al., 2015; Shi et al., 2017). Thus, we speculate that, similar to other diseases with disrupted structural networks as a common manifestation, the symptoms, and severity of PSD both relate to the level of disruption in the underlying white matter substrate.

### Limitations

Despite the novelty of the current study, this prospective research has several limitations. One important limitation is the fact that the research relied on deterministic tractography to reconstruct the whole brain structural network. This method has been shown to have limited capacity for resolving the crossing fiber issue. Future studies using a probabilistic tracking algorithm could complement our research. Secondly, at the current stage, we have not been able to determine the causal relationship between network alterations and neuropsychological symptoms. Thirdly, past studies have identified several risk factors for PSD, such as elevated 5-HTTLPR (Guo et al., 2016), serum levels of homocysteine (Li et al., 2017), and ferritin (Zhu et al., 2016). Unfortunately, we were unable to obtain any of these blood chemistry profiles to investigate the relationship between PSD and laboratory biomarkers. Future studies might consider including these blood test results to draw more comprehensive conclusions. A causal model might be beneficial to our understanding of the causal mechanisms behind depression. Finally, our study followed a cross-sectional design and was explorative in nature. Future longitudinal studies with additional post-treatment datasets might further improve our understanding of the potential role of network measures as biomarkers for treatment responses.

## Conclusion

In conclusion, we have successfully demonstrated global and local network reorganization in regions located in the limbic and cognitive systems of PSD patients compared to PSND patients. More importantly, our results suggest that both global and regional structural brain network topologies could potentially serve as indicators of the overall severity of PSD. These results contribute structural evidence for future research efforts seeking to understand the underlying neuroanatomical substrate behind the pathophysiology of PSD.

## Data Availability

Data are available upon reasonable request.

## Ethics Statement

This study was approved by the local Institutional Review Board, and informed consent was obtained from all participants.

## Author Contributions

XX and RT conducted the investigation and wrote the manuscript. LZ facilitated the patient recruitment and literature retrieval. ZC contributed to the study design and clinical data collection.

## Conflict of Interest Statement

The authors declare that the research was conducted in the absence of any commercial or financial relationships that could be construed as a potential conflict of interest.

## Footnotes

1 http://trackvis.org

2 http://www.R-project.org/
